# 2-Ethylhexanol Derivatives as Nonionic Surfactants: Synthesis and Properties

**DOI:** 10.1007/s11743-015-1760-0

**Published:** 2015-11-30

**Authors:** Wiesław Hreczuch, Karolina Dąbrowska, Arkadiusz Chruściel, Agata Sznajdrowska, Katarzyna Materna

**Affiliations:** MEXEO, 47-225 Kędzierzyn-Koźle, Poland; Department of Chemical Technology, Poznan University of Technology, 60-965 Poznan, Poland

**Keywords:** Nonionic surfactants, 2-Ethylhexanol, Surface activity, Ethoxylation, Propoxylation, Foaming, Homologue distribution

## Abstract

The synthesis and basic properties of 2-ethylhexanol based innovative nonionic surfactants are described in this paper. 2-Ethylhexanol as an available and relatively inexpensive raw material was used as the hydrophobe source modified by propoxylation and followed by polyethoxylation. As the result, six series of 2-ethylhexyl alcohol polyalkoxylates (EHP_*m*_E_*n*_) were obtained with three steps of propoxylation, each followed by polyethoxylation and two series only with polyethoxylation (EHE_*n*_). Two different catalysts were used, a dimetalcyanide and KOH. Values of average conversion rates and chemical content of the obtained products (GC, TG and GPC techniques) were compared. The influence of the applied catalyst and polyaddition degree on the homologue distribution, reactant conversion and amount of byproducts is discussed. The basic physicochemical parameters including refractive index, solubility in polar media, foaming properties and wettability were investigated and compared. Furthermore, surface activity parameters, i.e. surface tension (*γ*_CMC_) and critical micelle concentrations were determined. Results are compared to C_12–14_ alcohol ethoxylates (LaE_*n*_). Accordingly, it was found that the studied 2-ethylhexyl alcohol based compounds are effective, low foaming nonionic surfactants.

## Introduction

Surface active agents have a wide range of applications. Approximately 60 % of all surfactants are used in cleaning formulations, including household detergents, cosmetics, toiletry and hygiene products. Additionally, they are used in industrial cleaning (e.g. *in situ*) and as disinfection agents providing veterinary hygiene. The rest of surfactants are used in agrochemical and industrial applications where “detergency” is not so important, i.e., construction, coatings, inks, herbicides. Their global production reaches several million tons per year. Therefore, the impact of every new kind surfactant on humans and the environment must be investigated. However, it can be observed that producers and consumers are not willing to cover the increased costs of natural raw materials. This is exemplified by a surprisingly low market share of relatively expensive alkyl polyglucosides, in spite of their completely natural origin and good performance. Thus, one of the most important elements to develop and implement innovative surfactants is searching for alternative sources—raw materials at competitive prices, which meet the required safety and environmental criteria. An example of such a material is 2-ethylhexyl alcohol, which is widely available and relatively inexpensive, in comparison to C_12_–C_14_ alcohols. These fatty alcohols which are commonly used in the synthesis of surface active agents usually have between 11 and 18 carbon atoms per molecule. Generally, it is assumed that only then hydrophobic character of the hydrocarbon chain is sufficient for surfactants with suitable performance properties in the household and industrial applications for emulsification, wetting, washing or cleaning. However, effective implementations of C_10_ or even C_6_ hydrophobes for cleaning purposes were reported in the literature [1, 2].

2-Ethylhexanol (2-EH) is an oxo alcohol, mostly used in the production of phthalate plasticizers (approx. 70 %) and acrylate esters (approx. 20 %). As the result of a radical reduction in application of the 2-EH derived phthalates during the last decade, significant spare capacities of 2-ethylhexanol can be available for new applications at competitive prices.

2-Ethylhexanol polyoxyethylates have not been widely used as nonionic surfactants previously, because they are not amphiphilic enough. Their share in the production of polyoxyethylates is assessed to be <1 %, which may mean values near zero in practice [3].

Polyaddition of oxirane is usually carried out in the presence of alkaline catalysts, mostly sodium and potassium hydroxides. There are also known oxyalkylation catalysts, obtained from rare-earth metals, which provide a narrower range of homologue distribution and a relatively higher conversion of starter, as compared to the alkaline catalysts [4]. The alkoxylation reaction could include also the so called double metal cyanide (DMC) catalyst, which generates narrow range distribution of homologues. DMC type catalyst is much more reactive than KOH. These catalysts are salts composed of cation of a transition metal and an anion complex built of the second transition metal atom coordinated with the complexing anion CN^−^.

**Scheme 1:**
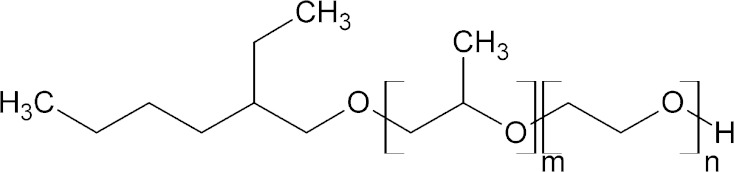
Structure of prepared compounds, where 0 ≤ *m* ≤ 3, 3 ≤ *n* ≤ 12 and *m*, *n* are the natural numbers

The aim of this work was to investigate basic characteristics of 2-ethylhexyl alcohol derivatives as nonionic surfactants synthesized in the presence of innovative DMC catalyst and compare them with two groups of nonionic surfactants, one obtained with the traditional catalyst KOH, and the other constituted by dodecyl alcohol ethoxylates. The studied series of products can be described by the following formula: EHP_*m*_E_*n*_, where 0 ≤ *m* ≤ 3, 3 ≤ *n* ≤ 12 and *n*, *m* are natural numbers, reflecting the average polyaddition degree of methyloxirane (P) and oxirane (E) (Scheme 1). The concept of the 2-EH based surfactants seems to respond complex environment, economic, and application requirements on the global market of detergents.

## Experimental Section

### Materials

The following chemicals were used as raw materials for the syntheses:

- 2-Ethylhexyl alcohol (molecular weight *M*_w_ = 130 g mol^−1^), ≥99.5 % pure, manufactured by Azoty Nitrogen Works in Kędzierzyn-Koźle, Poland.
- Oxirane (*M*_w_ = 44 g mol^−1^), 99.9 % pure, manufactured by PKN ORLEN S.A., Plock, Poland;
- Methyloxirane (*M*_w_ = 58 g mol^−1^), manufactured by PCC Rokita S.A., in Brzeg Dolny, Poland;
- Potassium hydroxide 85 %, analytical grade, purchased from Chempur, Piekary Śląskie, Poland;
- DMC-based catalyst manufactured by MEXEO Company in Kędzierzyn-Koźle, Poland;
- C_12_–C_14_ alcohol, acquired from PCC Rokita S.A., in Brzeg Dolny, Poland.

### Synthesis

Ethoxylation and propoxylation reactions were performed in a procedure as found in the literature [5]. The syntheses were carried out using a 1-dm^3^ autoclave, which is composed of automatic mixer, heating mantle and cooler. A pre-designed amount of alkylene oxide was introduced into the reactor charged with a weighed amount of starter and catalyst, following prior dehydration by a nitrogen purge at 120 °C for 20 min. The syntheses were conducted at 130 °C. Then, the final product was kept for another 60 min at the reaction temperature. At the end of synthesis, the product was cooled down and unloaded.

### Measurements

#### Gas Chromatography Determination

Gas chromatography was conducted using a Perkin Elmer GC apparatus composed of a flame-ionization detector. Separation was conducted under specified conditions. The column temperature was programmed in the range 70–300 °C with the temperature raised at a rate 8 °C min^−1^, the injector and detector temperatures were set at 300 and 400 °C, respectively. Argon was used as the carrier gas.

#### Thermogravimetric Measurements

Thermogravimetric analysis was performed using a Mettler Toledo Star^e^ TGA/DSC1 unit (Leicester, UK) under nitrogen. Samples between 2 and 10 mg were placed in aluminium pans and heated from 30 to 450 °C at a heating rate of 10 °C min^−1^.

#### Density

Measurements of density were carried out with Rudolph Research Analytical densimeter. The temperature was controlled and it was set up at 20 °C. Calibration was performed using deionized water and air as a reference substances. The uncertainty of the measurements was <10^−5^ g cm^−3^.

#### Refractive Index

Measurements of the refractive indices were conducted using a Rudolph Research Analytical refractometer. The temperature was controlled electronically and it was set at 20 °C. The uncertainty of measurements was <10^5^.

#### Hydroxyl Number

The specified amount of sample was weighed into a 250-cm^3^ Erlenmeyer flask and recorded to the nearest 0.0002 g. The specified amount of acetic anhydride–pyridine reagent was accurately pipetted into the flask, and a reflux condenser was attached. The flasks were placed in the 95–100 °C oil bath for 15 min. Next, the flasks were removed from the oil bath, cooled and 10 cm^3^ of deionized water was added. Using a dropper, 1 cm^3^ of phenolphthalein solution was added. The burette was filled and the sample was titrated using methanolic potassium hydroxide to a faint pink color. The hydroxyl value was calculated from the expression 56.1 *v/m,* where *v* is the difference, in cm^3^, between the two titrations and *m* is the quantity, in *g*, of the substance taken.

#### Cloud Point

Cloud point (CP) measurements of 1 % EHE_*n*_ and EHP_1_E_*n*_ butyldiglycol solutions (BDG) were determined and evaluated visually. The solution was heated until it fully clouded. Then the solution was cooled down with stirring until clarified. The cloud point was taken as the temperature at which the solution was completely transparent. CP values were averaged over six measurements.

#### Foaming

The measurements were determined using a 1-dm^3^ cylinder loaded with 200 cm^3^ 1 % solution of the sample. Then, 30 shakes for 30 s were performed. After shaking, the height of the produced foam (mm) was measured. The measurements were repeated after 1 and 10 min. Tests were performed three times for each sample.

#### Surface Tension

Surface tension measurements of the surfactant aqueous solutions were performed using a Drop Shape Analyzer from Krüss, Germany. The accuracy of each measurement was ±0.01 mN m^−1^. The temperature was set at 25 °C and controlled by a thermostatic bath. The drop shape method was used. Surface tension measurements were based on the camera image and calculated by analyzing the shape of the hanging drop using Laplace equation and specific software [6]. The values of the critical micelle concentration (CMC) were calculated using a linear regression analysis method.

#### Wettability

Measurements were carried out by the use of Drop Shape Analyzer DSA 100E (Krüss GmbH, Germany). The basis for the determination of the contact angle is the image of the drop on the examined surface (paraffin). After determination of actual drop shape and the contact line, the drop shape is adapted to fit a mathematical model used to calculate the contact angle. The most exact method to calculate this value is Young–Laplace fitting (sessile drop fitting). The complete drop contour is evaluated. After successful fitting of the Young–Laplace equation, the contact angle is determined as the angle between the solid/liquid and liquid/air phases.

## Results and Discussion

### Homologue Distribution

**Scheme 2:**
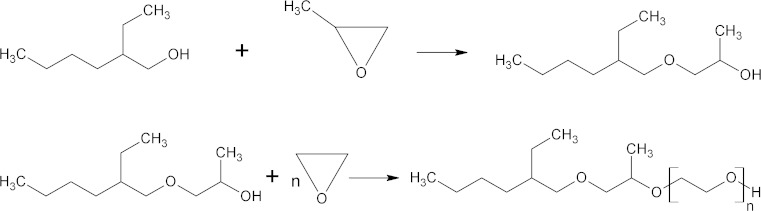
Scheme of the sequential propoxylation 2-ethylhexanol followed by polyethoxylation

Derivatives of 2-ethylhexyl alcohol were synthesized by the polyoxyalkylation reaction. The structure of the prepared compounds is presented in Scheme 1. Methyloxirane was added to 2-ethylhexanol in order to increase its hydrophobic character in the first step, followed by polyethoxylation, to obtain amphiphilic compounds (Scheme 2).

**Fig. 1:**
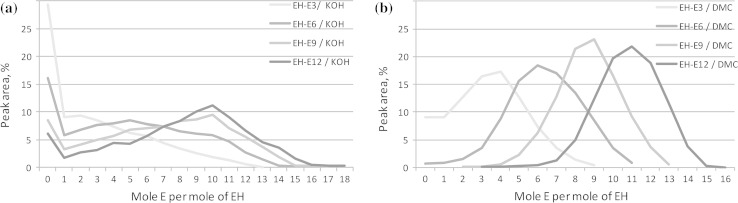
Comparison of homologue distributions determined by gas chromatography (GC) for 2-ethylhexanol ethoxylates of different average polyaddition degrees, as obtained with KOH (**a**) and the DMC type catalyst (**b**), respectively

Results on GC quantitative determination for the first series of 2-ethylhexanol ethoxylates (EHE_*n*_) are presented in figure. It can be seen from Fig. 1 that by application of the DMC type catalyst, the generated product distributions are narrower, as compared to KOH. It was widely reported that narrowing of homologue distribution improves physical and chemical properties, as well as environmental interactions, widening the application possibilities of ethoxylated products [7–9]. Moreover, significantly higher conversion of the starter alcohol is evidenced; this is due to very high activity of DMC-type catalysts, which is their major advantage in the polyaddition of oxiranes. This enables us to use them in a very small concentration (down to ppm). The formula of DMC catalysts can be described as follows:

1 $${\text{Me}}_{x}^{\text{I}} \left[ {{\text{Me}}^{\text{II}} \left( {\text{CN}} \right)_{6} } \right]_{y} \cdot L_{{a_{1} }}^{1} \ldots L_{{a_{n} }}^{n} , ,$$

where Me^I^ and Me^II^ denote transition metals (mostly Zn and Co), $L_{{a_{1} }}^{1} \ldots L_{{a_{n} }}^{n}$ mean suitable ligands (alcohols or ethers), and *x*, *y*, *a*_1_…*a*_*n*_ are integers. Generally, DMC catalysts are prepared by the reaction of a selected metal salt with a cyanide derivative of another metal (mostly alkaline) in the presence of an organic ligand. Regardless of the appropriate selection of substrates, preparation of the DMC catalysts is highly complex, with many critical factors, which determine the obtained catalytic activity and the quality of the polyadducts. The precise characteristic of DMC is described in one of our previous papers [10].

**Fig. 2:**
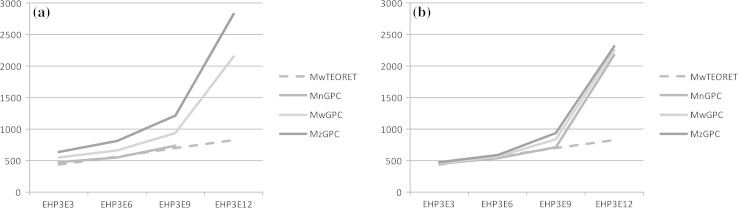
Example of the average molecular weights, as calculated from the GPC determinations, reflected by the *M*_*n*_, *M*_w_ and *M*_*z*_ parameters, for the EHP_3_E_*n*_ products obtained with KOH (**a**) and DMC (**b**) as catalysts, respectively

However, in spite of the differences of homologue distributions generated by the two compared catalysts, an additional question arises with regard to the quantities of byproducts. It had already been reported that parallel to the desired polyaddition, polymerization occurs and polyoxyethylene and polyoxypropylene diols are formed, especially at high molecular weights [11]. Therefore, size exclusion chromatography was applied to compare the products, depending on the average polyaddition degrees and the applied catalyst. It can be seen from Fig. 2, that the average molecular weights determined by GPC for the DMC-based products are close to the theoretical ones, as calculated from the synthesis weight balance. For that purpose the *M*_w_, *M*_*n*_ and *M*_*z*_ parameters were calculated from the GPC chromatograms by the following equations:

2 $$M_{n} = \frac{{\sum N_{i} M_{i} }}{{\sum N_{i} }};\,M_{w} = \frac{{\sum N_{i} M_{i}^{2} }}{{\sum N_{i} M_{i} }};\,M_{z} = \frac{{\sum N_{i} M_{i}^{3} }}{{\sum N_{i} M_{i}^{2} }};$$

where *M*_*i*_ is molar mass of an *i*th individual molecule and *N*_*i*_ is the number of particles *i* in the sample. More significant differences among the *M*_w_, *M*_*n*_ and *M*_*z*_ parameters were found in the case of the KOH based products.

Larger gaps between the average molecular weights determined by GPC and the theoretical weight balance were noted at the average polyaddition degree (*N*_av_) equal to 12. Even at *N*_av_ = 9, KOH yields already remarkable discrepancy. It means that along the increasing molecular weight, the polymerization rate overcomes that of polyaddition. It may be explained by formation of polymers (diols) with two reactive centers at each chain and double consumption of oxirane, relative to the monofunctional polyadducts. Furthermore, the alkaline catalyst was found to be less selective for the products with higher average propoxylation degree, which seems neutral in the case of DMC, where the calculated *M*_w_, *M*_*n*_ and *M*_*z*_ values were mostly close each to the other (Fig. 2).

Additionally, the homologue distribution can also be compared by the polydispersity parameter MWD = *M*_w_/*M*_*n*_. The calculated MWD distribution parameters for the studied series of EHP_*m*_E_*n*_ obtained with KOH and DMC as the catalysts are presented in Table 1.

**Table 1:** Physical and chemical properties of synthesized surfactants in comparison to nonionic C_12_–C_14_ alcohol surfactants

Compound	Density^a^ (g cm^−3^)	Rf^b^	Cloud point^c^ (°C)	Rp^d^	MWD^e^	*T*_onset50_^f^ (°C)	State^g^
KOH							
EHP1E3	0.917	1.426	–	0.40	1.19	209	Liquid
EHP1E6	0.963	1.433	47.4	0.70	1.15	341	Liquid
EHP1E9	1.021	1.443	76.2	1.50	1.15	385	Liquid
EHP1E12	–	1.452	–	7.50	1.09	411	Solid
EHP2E3	0.945	1.430	38.1	1.12	1.12	261	Liquid
EHP2E6	0.989	1.436	62.7	1.15	1.15	342	Liquid
EHP2E9	–	1.445	81.8	1.11	1.11	390	Wax
EHP2E12	–	1.453	94.5	1.09	1.09	402	Solid
EHP3E3	0.958	1.430	36.2	0.70	1.14	285	Liquid
EHP3E6	1.002	1.439	63.5	0.80	1.19	375	Liquid
EHP3E9	–	1.445	84.3	1.30	1.15	400	Wax
EHP3E12	–	1.453	95.5	2.30	1.08	405	Solid
EHE3	0.958	1.431	41.6	1.30	1.11	291	Liquid
EHE6	1.010	1.439	69.9	1.50	1.12	351	Liquid
EHE9	–	1.444	82.1	2.70	1.13	370	Wax
EHE12	–	1.446	88.2	1.50	1.16	399	Solid
LaE3	0.930	1.435	45.3	–	–	–	Liquid
LaE6	0.958	1.440	71.4	–	–	–	Liquid
LaE9	–	1.443	82.1	–	–	–	Wax
LaE12	–	1.446	88.5	–	–	–	Solid
DMC							
EHP1E3	0.915	1.426	–	17.5	1.10	220	Liquid
EHP1E6	0.963	1.434	53.9	25.0	1.06	340	Liquid
EHP1E9	1.021	1.443	80.0	39.5	1.05	384	Liquid
EHP1E12	–	1.452	89.6	147.4	1.04	403	Solid
EHP2E3	0.941	1.429	37.5	17.7	1.05	269	Liquid
EHP2E6	0.983	1.429	61.0	24.3	1.05	343	Liquid
EHP2E9	1.035	1.445	82.1	96.3	1.05	391	Liquid
EHP2E12	–	1.454	96.2	93.7	1.04	398	Solid
EHP3E3	0.954	1.432	42.4	26.6	1.04	302	Liquid
EHP3E6	0.997	1.439	66.1	33.1	1.05	365	Liquid
EHP3E9	–	1.446	88.1	56.9	1.16	397	Wax
EHP3E12	–	1.455	94.0	107.2	1.03	400	Solid
EHE3	0.957	1.430	50.9	24.3	1.10	278	Liquid
EHE6	1.003	1.438	72.2	19.3	1.05	350	Liquid
EHE9	1.031	1.443	85.5	16.8	1.05	388	Liquid
EHE12	1.048	1.447	90.3	13.2	1.08	391	Liquid
LaE3	0.932	1.435	55.6	–	–	–	Liquid
LaE6	0.950	1.440	77.4	–	–	–	Liquid
LaE9	–	1.444	82.8	–	–	–	Wax
LaE12	–	1.447	89.5	–	–	–	Solid

^a^At 20 °C

^b^At 60 °C

^c^Values of cloud point in 25 % butyldiglycol (BDG) solution at 25 °C

^d^Rp average reactivity parameter

^e^MWD polydispersity parameter

^f^ *T*_onset50_ decomposition of 50 % of the sample

^g^At 20 °C

It can be observed that the determined MWD values for products synthesized in the presence of DMC catalyst are lower, as compared to their equivalents produced with KOH. This can also confirm a more selective reaction towards the aimed molecular weights with DMC type catalyst, where the concentration of homologues near the average polyaddition degree is higher.

The much more complex homologue distributions of the EHP_*m*_E_*n*_ series are difficult to determine quantitatively by the GC technique. Generally, polydispersed products of narrower distributions should contain lower amounts of the unconverted starter, as well as lower concentrations of the low molecular fractions. As the result, they must be assessed by relevant differences determined by thermogravimetric (TG) measurements, where the lower fractions evaporate at lower temperatures. Such an approach had already been reported in the literature [12].

Thermal analysis shows that the decomposition temperatures (*T*_onset50_) for EHP_*m*_E_*n*_ based on KOH are in the range 209–411 and 220–403 °C for DMC ethoxylates. Note that the greater average propoxylation grade (P_1_–P_3_) is, the higher the temperature of mass loss. The same can be observed with the polyethylene ether chain (E_3_–E_12_) length. However, the role of propoxylation was found to be negligible, because temperatures of decomposition for EHE_*n*_ were in a similar range to EHP_*m*_E_*n*_. The EHP_3_E_12_ products at 50 % mass reduction already fall as a whole within the temperature range where thermal decomposition dominates, so the observed differences in mass decrease between the KOH and DMC equivalents are reduced. The discussed relationship for all series of the studied products is presented in Table 1.

**Fig. 3:**
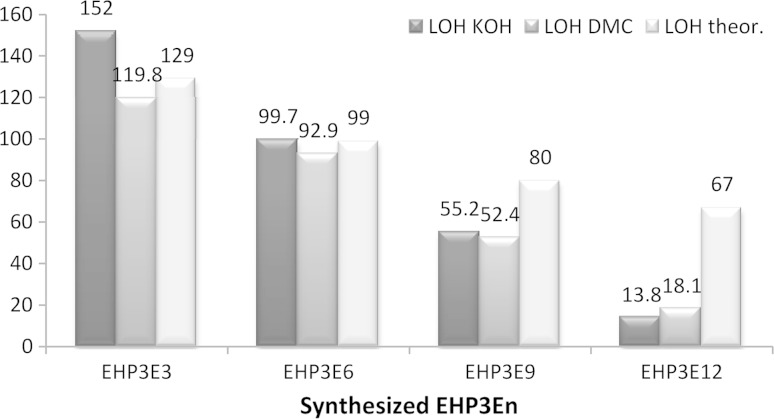
Comparison of hydroxyl numbers determined experimentally and calculated theoretically, depending on the average ethoxylation degrees (*N*_av_)

The same was also confirmed by determination of hydroxyl numbers (*L*_OH_) for the discussed series of products, where the experimental *L*_OH_ values indicate much higher molecular weight then the theoretical values calculated from the synthesis weight balance, especially for the products with *n* = 9 and *n* = 12 (Fig. 3).

Concentrations of DMC and KOH catalyst at 130 °C were 0.05 and 0.3 wt% per product weight, respectively. Therefore, the average reactivity parameter, Rp, was calculated to compare the unit conversion rates in each case:

3 $$Rp = \frac{{g_{A} }}{{g_{\text{C}} \times g_{\text{EH}} \times h}}$$

where *g* means mass (grams), *A* is methyloxirane or oxirane, EH states for 2-ethylhexanol, *h* denotes hour and *C* means catalyst.

It can be seen from Table 1, that the DMC type catalyst is much more reactive than KOH. Both, propoxylation and ethoxylation commenced many times faster, which is desired from economical point of view, especially if the product quality is equivalent or better.

### Physicochemical Properties

#### Refractive Index and Hydroxyl Number

The refractive index (Rf) was determined for the studied series of products to investigate their sensitivity towards the average polyaddition degree and homologue distribution. Values were determined at 60 °C, where all of the investigated samples were liquid (Table 1). Values of Rf index increase with the average polyaddition degree. Therefore, not a big difference was found between the series of products generated with DMC and KOH as catalysts. However, it is interesting that the addition of the first propoxy-mere decreases the values of Rf index, as compared to the EHE_*n*_ series. It may be a steric effect of the methyl branched chain. At higher average polyaddition degrees (*n* > 6) the discussed influence of the first P block is dominated by the larger number of the additive ether bounds of the ethoxy blocks.

The complex analytical investigation by GC, TG, GPC techniques supplemented by determination of *L*_OH_ numbers and Rf indexes gives a clear picture of the composition of the studied products. It was proven that they are distinguished by a much narrower homologue distribution and selectivity where the DMC catalyst is applied. The differences are significant, especially at lower average polyaddition degrees (*n* < 9). At the higher range of molecular weight (*n* > 9) the polyaddition reaction seems dominated by parallel polymerization yielding larger amounts of the polymer diols.

#### Cloud Point

One of the key features of surfactants is their solubility in polar media. Values of the cloud point in 25 % butyldiglycol (BDG) solution were determined (Table 1). Again, the recorded solubility of the studied surfactants in polar solutions (BDG) indicates similar behavior of the investigated EHP_*m*_E_*n*_ series compared to those of C_12–14_ alcohol. The temperatures of the cloud point rise in the series of homologues for both catalysts, i.e. values for EHP_2_E_*n*_ are in the ranges 38.1–94.5 and 38.1–94.5 °C for KOH and DMC derivatives, respectively. The temperatures increase with the length of polyoxyethylene ether chain in both groups. Among P_1_–P_3_ blocks there are some differences, but not so significant, i.e. 80.0 °C for EHP_1_E_9_, 82.1 °C for EHP_2_E_9_ and 88.1 °C for EHP_3_E_9_ in the DMC group. The same minor differences are observed for compounds derived using KOH.

The results from Table 1 show that with the elongation of the polyoxyethylene chain the physical state changes. Compounds EHP_*n*_E_3_, EHP_*n*_E_6_ and EHP_1_E_9_ based on both catalysts are liquids. However, DMC-derived EHP_2_E_9_ is a liquid, whereas the KOH-derived one is a solid. Physical states of compounds from the EHP_3_E_*n*_ group for both catalysts do not differ much. Ethoxylates with higher polyaddition degree, where *n* = 12, are solid. However EHE_*n*_ DMC products remain less viscous and liquid at higher polyaddition degrees in comparison to KOH based ethoxylates. The liquid state is much more convenient from the technological point of view, because the material does not require melting for transport and discharge. Generally, the EHP_*m*_E_*n*_ products appear very similar to their C_12_–C_14_ alcohol equivalents, in this aspect. A higher average propoxylation grade (P_1_–P_3_) tends to favour solidification at room temperature of the products of higher molecular weights. Physical and chemical results presented in Table 1 confirm that the EHP_*m*_E_*n*_ surfactants show a behavior similar to conventional C_12–14_ alcohol ethoxylates, which is a positive prerequisite for the market.

### Surface Activity

The surface-active properties of the series of 2-ethylhexyl alcohol derivatives are summarized in Table 2. One of the criteria of surface activity, characteristic for surfactants is the critical micelle concentration (CMC), defining their concentration in water solution, at which monomers start to aggregate into micelles. The CMC was determined for the water solutions of studied surfactant series and the results are shown in Table 2. The addition of methyloxirane decreases values of the CMC, while the lowest CMC is observed for the EHP_*m*_E_3–6_ systems. For these surfactants, CMC values are usually one order lower in comparison to EHE_*n*_. This supports the concept of enhancement of surface activity of the EH-based surfactants by the addition of the P_1_–P_3_ blocks into the molecule. Moreover, they appear close to those of the reference LaE_*n*_ surfactant series.

**Table 2:** Surface activity of synthesized surfactants with comparison to nonionic C_12_–C_14_ alcohol surfactants

Compound	CMC (mmol dm^−3^)	*γ*_CMC_ (mN m^−1^)	*Γ*_max_ × 10^6^ (mol m^−2^)	*A*_min_ × 10^19^ (m^2^)	−Δ*G*_ads_^0^ (kJ m^−1^)	pC_20_	*Π*_CMC_ (mN m^−1^)	CA (°)
KOH								
EHP1E3	15.8	29.8	2.37	6.99	26.6	3.20	42.6	35.4
EHP1E6	10.0	27.6	2.72	6.10	27.7	3.20	44.8	35.7
EHP1E9	11.7	29.5	2.31	7.20	28.0	3.51	42.9	46.6
EHP1E12	44.7	39.4	1.46	11.3	27.9	2.60	33.0	70.3
EHP2E3	3.09	30.6	2.53	6.56	30.1	3.81	41.8	31.6
EHP2E6	5.62	28.0	2.87	5.79	28.2	3.51	44.4	33.8
EHP2E9	10.0	30.3	3.17	5.24	24.6	3.20	42.1	50.1
EHP2E12	85.1	40.1	1.10	15.1	32.0	2.60	32.3	70.4
EHP3E3	6.46	30.0	3.20	5.20	25.6	3.51	42.4	40.2
EHP3E6	6.92	28.4	3.06	5.43	26.6	3.51	44.0	37.7
EHP3E9	12.9	29.3	2.58	6.45	26.9	3.51	43.1	49.9
EHP3E12	79.4	39.1	0.87	19.2	35.3	2.30	33.3	75.8
EHE3	24.0	28.0	3.25	5.11	22.4	2.90	44.4	36.3
EHE6	28.8	26.9	2.69	6.17	24.1	2.90	45.5	39.5
EHE9	33.9	29.3	2.56	6.48	23.7	2.90	43.1	46.4
EHE12	38.9	31.1	2.24	7.40	24.3	2.60	41.1	57.2
LaE3	10.0	27.6	4.47	3.71	22.4	3.30	45.2	31.7
LaE6	0.21	27.6	5.77	2.88	29.5	4.71	45.2	33.1
LaE9	0.16	33.6	3.79	4.38	31.6	5.01	39.2	46.8
LaE12	0.16	38.3	3.36	4.95	31.6	4.71	34.5	56.9
DMC								
EHP1E3	25.1	30.6	3.31	5.02	21.5	2.90	41.8	48.2
EHP1E6	4.90	28.8	3.21	5.17	26.7	3.81	43.6	36.9
EHP1E9	11.7	31.0	2.60	6.39	26.6	3.51	41.4	50.6
EHP1E12	35.4	34.8	2.81	5.91	22.2	2.60	37.6	68.2
EHP2E3	3.71	30.6	2.60	6.38	29.4	3.81	41.8	37.2
EHP2E6	5.75	28.4	2.71	6.12	28.7	3.81	44.0	38.3
EHP2E9	10.7	31.4	2.14	7.75	29.7	3.81	41.1	52.3
EHP2E12	40.7	33.3	2.60	6.39	22.6	2.60	39.1	56.6
EHP3E3	2.40	30.9	2.53	6.55	31.3	4.11	41.5	40.6
EHP3E6	4.79	29.1	2.57	6.47	30.0	4.11	43.4	44.4
EHP3E9	11.5	31.6	1.91	8.68	31.2	3.81	40.8	57.8
EHP3E12	30.9	34.4	1.95	8.52	26.0	2.90	38.0	70.5
EHE3	16.2	27.6	3.33	4.99	23.2	3.20	44.9	34.9
EHE6	20.9	29.7	2.89	5.75	23.8	3.20	42.7	41.2
EHE9	29.5	35.3	2.12	7.84	25.3	2.90	37.1	55.4
EHE12	33.1	37.8	1.94	8.56	25.5	2.90	34.7	64.8
LaE3	0.12	28.2	12.2	1.36	25.4	4.51	44.6	33.8
LaE6	0.15	30.8	5.49	3.02	29.2	4.61	42.0	41.2
LaE9	0.10	33.8	5.07	3.27	29.8	4.71	39.0	53.9
LaE12	0.16	39.7	3.09	5.37	32.1	4.56	33.1	68.5

Furthermore, the values of the surface tension *γ*_CMC_ of the investigated surfactants were determined (Table 2).

**Fig. 4:**
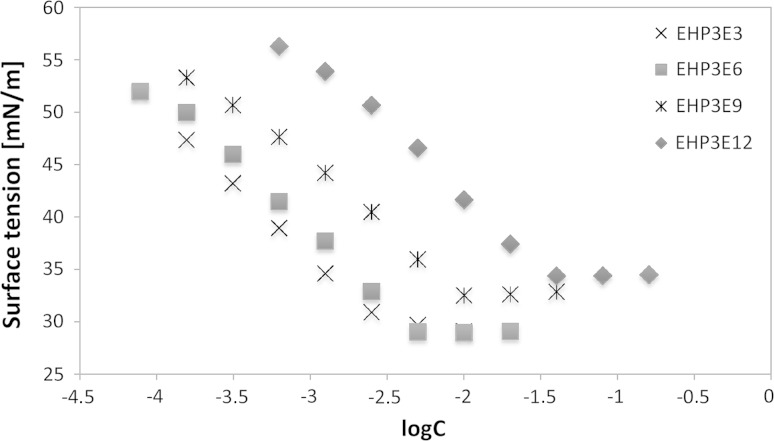
Surface tensions as a function of concentration (log*C*) for EHP_3_E_*n*_ based on the DMC catalyst

The surface tension of aqueous solutions *γ*_CMC_ of the studied surfactants decreased from water value (72.8 mN m^−1^) to a minimum located from 26.9 to 40.07 mN m^−1^, where it reached a plateau. The determined surface tensions for P_1_–P_3_ blocks appear at the similar level of common values. The influence of homologue distribution in EHP_3_E_*n*_ group, catalyzed with DMC is presented in Fig. 4. Generally, values of surface tension *γ*_CMC_ increase with the length of polyethylene ether chain. However, the curves represented compounds with low polyaddition degree, where *n* = 3 and *n* = 6, show that values of *γ*_CMC_ and CMC do not differ significantly.

Calculations of surface excess concentrations *Γ*_max_, Gibbs free energy of the adsorption layer Δ*G*_ads_^0^ and the minimum surface area occupied by a molecule at the interface *A*_min_ were described earlier [13]. It was observed that values of these parameters do not differ significantly. However, with the elongation of polyethylene ether chain, the values of *A*_min_ for EHP_1_E_*n*_ increase from 6.99 to 11.3 × 10^−19^ m^2^. This may be caused by hydration of the polyethylene ether chain. It was found that the length of the polyethylene ether chain determines the size of the surface area occupied by a molecule. The negative values of Δ*G*_ads_^0^ for all studied surfactants indicate that the process proceeds spontaneously [14].

There are two additional surface activity parameters, the adsorption efficiency, pC_20_, and the effectiveness of surface tension reduction, *Π*_CMC_, which are calculated from surface tension measurements. The pC_20_ is defined as the negative logarithm of the surfactant concentration in the bulk phase required to reduce the surface tension of the water by 20 mN m^−1^, which represents efficiency of surface adsorption on an air–water interface. Then, the greater the pC_20_ value is, the higher is the adsorption efficiency of the surfactant. The other parameter, *Π*_CMC_ is the surface pressure at the CMC, being defined by $\varPi_{\text{CMC}} = \gamma_{0} - \gamma_{\text{CMC}} ,$ where *γ*_0_ is the surface tension of pure solvent and *γ*_CMC_ is the surface tension of the solution at the CMC. Surface pressure at the CMC provides information on how significant is the ability of the surfactant to reduce the surface tension of the solvent and hence, what is the effectiveness of this phenomenon. It can be observed that the highest values of pC_20_ were obtained for EHP_2_E_3_ KOH and EHP_3_E_3–6_ DMC, which is equal to 3.81 and 4.11, respectively. The highest effectiveness of surface tension reduction *Π*_CMC_ was determined for EHE_6_ KOH and EHE_3_ DMC, 45.5 and 44.9 mN m^−1^, respectively. Moreover, these values are comparable to those from LaE_*n*_ (Table 2).

**Fig. 5:**
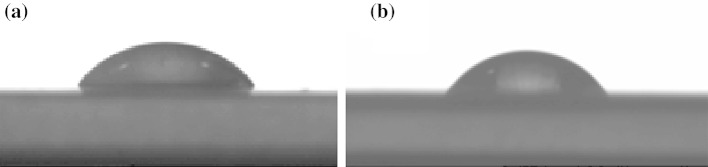
Drops of 2-ethylhexyl alcohol derivative on the paraffin surface EHE_12_ KOH (**a**) and C_12_–C_14_ alcohol equivalent LaE_12_ KOH (**b**)

Wettability was also investigated for the studied surfactant series and the results of contact angle measurements (CA) are presented in Table 2. Values of CA increase with the length of polyoxyethylene chain. There are no significant differences in CA measurements between EHE_n_ and their nonionic equivalents LaE_*n*_. This similarity is best illustrated in Fig. 5, where the drops of EHE_12_ (57.2°) and its nonionic equivalent LaE_12_ (56.9°) are compared. Reduction of CA is essential for the cleaning or washing effect, because of better wetting of hydrophobic surfaces.

Generally, there are no significant differences in surface-active parameters for EHP_*m*_E_*n*_ and EHE_*n*_ in comparison to their nonionic equivalents LaE_*n*_.

**Fig. 6:**
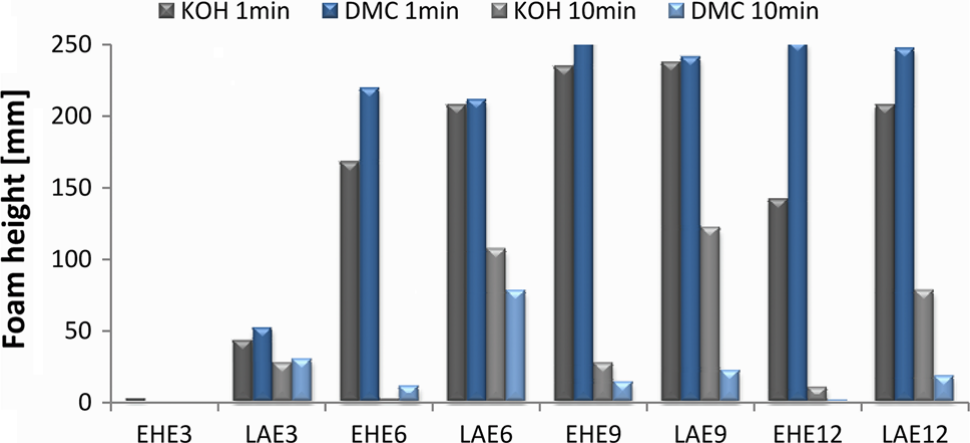
Exemplary comparison of foaming for the studied EHE_n_ surfactant series and their C_12_–C_14_ alcohol equivalents

A practically important issue of surfactant solutions is their foaming performance, which is required at a high or minimum level depending on application. Many reports on foamability of polyglycerol fatty acid ester are available [15–18]. The most common detergent applications include the household automotive washing or industrial Clean in Place systems (CIP), which require minimum or not foaming at all. The other bulk applications like lubrication, flotation and many others do also limit foaming of the applied surfactants. The example of foaming performance for the studied series of surfactants and their nonionic equivalents is presented in Fig. 6. It might be noted, that while the initial foaming (after 1 min observation) is comparable for most of the studied nonionic series, the stability of foam (after 10 min) of the EHE_*n*_ is visibly lower than LaE_*n*_. It may point to the application of EHE_*n*_ (especially those synthesized with DMC catalyst) as low-foaming surfactants.

## Conclusions

Six series of 2-ethylhexanol alkoxylates were investigated as innovative surfactants, represented by a general formula EHP_*m*_E_*n*_, where *m* is a natural number within the range 0 ≤ *m* ≤ 3; surfactants were obtained with a KOH and a DMC-type catalyst, respectively. Each of the series included ethoxylation products of average polyaddition degree (*n*) equal to 3, 6, 9 and 12.

It was confirmed that the DMC-type catalyst provides significantly narrower distribution of homologues and higher conversion of the starting material. Additionally, higher selectivity and lower content of byproducts was also evidenced in the case of the DMC-type catalyst as compared to KOH.

The performed studies show that the DMC and KOH-derived EHP_*m*_E_*n*_ surfactant series exhibit comparable physicochemical properties, as well as interfacial performance similar to that of reference C_12_–C_14_ alcohol ethoxylates. Generally, the concept of insertion of P_*m*_ blocks into the EHE_*n*_ molecule was positively confirmed by its influence on relative decrease of the CMC values. Moreover, the values of minimum surface area *A*_min_ increase with the elongation of polyethylene ether chain. This could be caused by the steric effect of the branched hydrophobes, as compared to the linear LaE_*n*_ products, which can adsorb vertically at the interface. Additionally, foam stability of the EHP_*m*_ based ethoxylates was lower, as compared to that of their LaE_*n*_ equivalents. EHP_*m*_E_*n*_ alkoxylates exhibit typical properties of low foaming nonionic surfactants and most of the physicochemical properties and surface activity is similar to that of the La-based surfactants.

Summarizing, EHP_*m*_E_*n*_ alkoxylates obtained using a cheap and accessible raw material have a high potential for application. Furthermore, the sterically specific structure of their hydrophobic moiety is very interesting. Therefore, this will be a subject of our further studies in more complex formulations.
